# PERINATAL CARE IN A NORTHEASTERN BRAZILIAN STATE: STRUCTURE, WORK PROCESSES, AND EVALUATION OF ESSENTIAL NEWBORN CARE COMPONENTS

**DOI:** 10.1590/1984-0462/2021/39/2019334

**Published:** 2020-11-20

**Authors:** Rafaella Willig de Quadros, Lara Damiani Cabral, Chaiana Esmeraldino Mendes Marcon

**Affiliations:** a Universidade do Sul de Santa Catarina, Tubarão, SC, Brazil.

Tubarão, January 29, 2020.

To: Revista Paulista de Pediatria

The article by Bezerra et al. reveals significant distortions regarding perinatal care in Sergipe. Despite showing data above the national average on aspects such as the presence of an on-duty pediatrician 24 hours a day and the availability of equipment for maternal and neonatal resuscitation in all maternity hospitals, the state still has deficits in several essential newborn care (ENC) measures.^1^

With respect to ENC components, the presence of companions from prenatal to postpartum was observed in only 18% of cases, and early skin-to-skin contact was noted in 41% of deliveries. However, only 33.1% of mothers breastfed in the first hour of life. Therefore, this letter aims at providing data from other Brazilian states in order to encourage improvements in actions for newborn care proposed by the World Health Organization (WHO).^1^

We emphasize that the reality of the Brazilian Northeast concerning ENC principles differs from the situation observed in the Southern region of the country, as shown in the study by Velho et al. The authors found that 88.1% of pregnant women were accompanied at all times by a person of their choice during delivery and that 51.8% of them had skin-to-skin contact with their child. This scenario was highlighted by a model of best practices in obstetric care, which promotes increased humanization during childbirth, resulting from the adoption of a greater proportion of evidence-based practices.^2^

Nevertheless, breastfeeding in the first hour after birth is still unsatisfactory throughout Brazil - rates similar to those of Sergipe (33.1%) - , not following the guideline proposed by the Ministry of Health that recommends this practice, which alone is the strategy that most prevents infant deaths. In the first hour of life, the child is in a quiet-alert state - quiet but alert and with eyes wide open - , as if paying attention.^3^

In the state of Sergipe, physical and structural care is considered adequate, but the ENC components are below those proposed by the WHO. The poor prevalence of breastfeeding in the first hour of life portrays the reality not only of this Northeastern state but also of Brazil.^1^

Given the seriousness of the above issues, measures to encourage the humanization of perinatal care must be implemented for neonatal and maternal benefits.
