# Supplementary material for: PERINATAL CARE IN A NORTHEASTERN BRAZILIAN STATE: STRUCTURE, WORK PROCESSES, AND EVALUATION OF ESSENTIAL NEWBORN CARE COMPONENTS
Source: Rev Paul Pediatr. 2020 Nov 20;39:e2019334. doi: 10.1590/1984-0462/2021/39/2019334 (PMC7681944; doi:10.1590/1984-0462/2021/39/2019334)
Supplement: Related texts

# Reply: AUTHOR’S RESPONSE LETTER

**Authors:** Felipa Daiana Bezerra, Maria Alexsandra da Silva Menezes, Rosemar Barbosa Mendes, José Marcos de Jesus Santos, Debora Cristina Fontes Leite, Samir Buainain Kassar, Ricardo Queiroz Gurgel

**Affiliations:** a Universidade Federal de Sergipe, Aracaju, SE, Brazil.; b Universidade Federal de Sergipe, Lagarto, SE, Brazil.; c Universidade Tiradentes, Aracaju, SE, Brazil.; d Universidade Estadual Ciências da Saúde de Alagoas, Maceió, AL, Brazil.

Dear colleagues, we are delighted to receive your comments on our article. We agree that Sergipe, like most of Brazil, falls short when it comes to the practices that make up ENC.

One of the paradoxes presented in our article is the presence of an adequate hospital structure for perinatal care (equipment for resuscitation and pediatricians in the delivery room, for example) associated with the low frequency of breastfeeding in the first hour of life and early skin-to-skin contact.^1^

Another study of national data that evaluated ENC practices highlighted the negative effect of cesarean sections on early skin-to-skin contact and breastfeeding in the first hour of life, regardless of maternal characteristics and hospital structure. The high rates of cesarean section indeed seem to contribute to the delay in mother-newborn contact. This finding is unfortunate, as these measures are important for reducing infant mortality.

These data emphasize the urgent need for changes in pregnancy, labor, and delivery care. Another significant result is the low adherence to ENC components in Sergipe, compatible with results found in the North and Northeast regions.^2^ In Brazil, the scenario is similar, but with regional disparities. A study by Velho et al. concluded that, just as in Sergipe, interventional obstetric care still predominates in the South region, with high rates of cesarean sections, but with greater adherence to ENC practices in public hospitals.^3^

As the frequency of cesarean delivery in Brazil remains high, changing the perinatal care model currently practiced is crucial. The WHO recommendations related to ENC are simple and inexpensive. They also have a positive effect on reducing neonatal mortality, showing that the implementation of best practices is closer to the commitment of multidisciplinary teams in wanting to change the Brazilian perinatal scenario than to the investment in technologies.

## References

[1] Bezerra FD, Menezes MA, Mendes RB, Santos JM, Leite DC, Kassar SB (2019). Perinatal care in a northeastern Brazilian state: structure,
work processes, and evaluation of the components of essential newborn
care. Rev Paul Pediatr.

[2] Menezes MA, Gurgel R, Bittencourt SD, Pacheco VE, Cipolotti R, Leal MC (2018). Health facility structure and maternal characteristics
related to essential newborn care in Brazil: a cross-sectional
study. BMJ Open.

[3] Velho MB, Brüggemann OM, McCourt C, Gama SG, Knobel R, Gonçalves AC (2019). Obstetric care models in the Southern Region of Brazil and
associated factors. Cad Saude Publica.
